# Comparative assessment of faecal microbial composition and metabonome of swine, farmers and human control

**DOI:** 10.1038/s41598-020-65891-4

**Published:** 2020-06-02

**Authors:** Shiang Chiet Tan, Chun Wie Chong, Ivan Kok Seng Yap, Kwai Lin Thong, Cindy Shuan Ju Teh

**Affiliations:** 1 Institute of Biological Science, Faculty of Science, University of Malaya, 50603 Kuala Lumpur, Malaysia; 2 School of Pharmacy, Monash University Malaysia, Jalan Lagoon Selatan, 47500 Bandar Sunway, Malaysia; 3 Centre for Translational Research, Institute for Research, Development and Innovation (IRDI), International Medical University, 57000 Kuala Lumpur, Malaysia; 4 Sarawak Research and Development Council, 11th Floor LCDA Tower, The Isthmus, Off Jalan Bako, 93050 Kuching, Sarawak Malaysia; 5 NANOCAT Research Centre, University of Malaya, 50603 Kuala Lumpur, Malaysia; 6 Department of Medical Microbiology, Faculty of Medicine, University of Malaya, 50603 Kuala Lumpur, Malaysia

**Keywords:** Microbiology, Bacteria, Microbial communities

## Abstract

The gastrointestinal tract of humans and swine consist of a wide range of bacteria which interact with hosts metabolism. Due to the differences in co-evolution and co-adaptation, a large fraction of the gut microbiome is host-specific. In this study, we evaluated the effect of close human-animal interaction to the faecal metagenome and metabonome of swine, farmer and human control. Three distinct clusters were observed based on T-RFLP-derived faecal microbial composition. However, 16S-inferred faecal microbiota and metabolic profiles showed that only human control was significantly different from the swine (P < 0.05). The metabonome of farmers and human controls were highly similar. Notably, higher trimethylamine N-oxide (TMAO) and butyrate were detected in human control and swine, respectively. The relative abundance of TMAO was positively correlated with *Prevotella copri*. Overall, we compared and established the relationship between the metabolites and microbiota composition of swine, farmers and human control. Based on the data obtained, we deduced that long term occupational exposure to swine and farm environment had affected the gut bacterial composition of farmers. Nonetheless, the effect was less prominent in the metabolite profiles, suggesting the gut bacteria expressed high functional plasticity and are therefore resilience to the level of community shift detected.

## Introduction

Gastrointestinal tract of humans and animals harbours a vast community of microorganisms which holds enormous physiochemical and metabolic potentials. The bacterial communities are able to interact with the diet, immune responses, genetic and epigenetic composition of the hosts by compensating numerous biological activities lacking in the host’s biological systems^1–3^. For instance, independent studies carried out using germ-free mice and human volunteers showed that through bacterial fermentation, gut microbiota are able to assist energy harvesting from diet and poorly digestible polysaccharides^4,5^. In addition, gut microbial community can also influence hosts’ neural development, cognition and behaviour^6^. It is therefore important to prevent the disruption of gut microbiome to maintain the stability of its functions.

Modulation of gut microbiome can also occur in response to external factors such as environmental stress, antibiotic treatments, diets and exposure to different groups of environmental bacteria^3,7^. In recent years, it is increasingly recognised that the interaction between humans, animals and their shared environments is an important determinant for public health. Such “One Health” concept has become more important amid the rise of industrial animal production which increased the proximity of the living space between humans and farm animals. For example, long term occupational interactions between humans and swine in swine farms may facilitate the transmission of anthropozoonoses and zooanthroponoses between humans and swine, especially diseases that can be found in both humans and animals such as rabies, brucellosis, salmonellosis and H1N1 virus. For instance, a surge in the prevalence of phylogenetic closely related strains of hepatitis E virus of swine and humans has been reported in animal reservoirs from Uruguay^8^.

Other than pathogenic microorganisms, an exposure to same microbial source may also results in the reciprocal exchange of non-pathogenic microbial community^9,10^. Studies had shown that young children who live or being raised in farm environment harbour a wide spectrum of microbes that confers certain degree of protection against the development of asthma and allergies^11–13^. Separately, the usage of antibiotics in the farm may also impact the commensals in humans and animals while increasing the establishment of antimicrobial resistant bacteria in gastrointestinal tract^14^. To date, many studies on swine-related metagenomics and metabolomics have been carried out mainly to improve the breeding strategies such as animal health assessment, bioproduct characterization, feed efficiency and livestock growth potential^15–17^. Comparatively, few had applied the One Health concept into the microbiome and metabonome to understand the interaction/transmission of gut microbiomes across hosts^18^.

In this study, we investigated the faecal metagenome and metabonome of swines and swine farmers. To elucidate the influence of farm environment to the gut microbial composition, human subjects who have no direct contact/access to the swine farm was selected as control. By comparing the metagenomics and metabolites profiles of these three groups, our study aimed to understand the interaction between human and animal microbiomes, and its impact to the host metabolisms.

## Results

### Comparison of faecal bacterial composition of swine, farmer and human control group based on T-RFLP analyses

**Figure 1:**
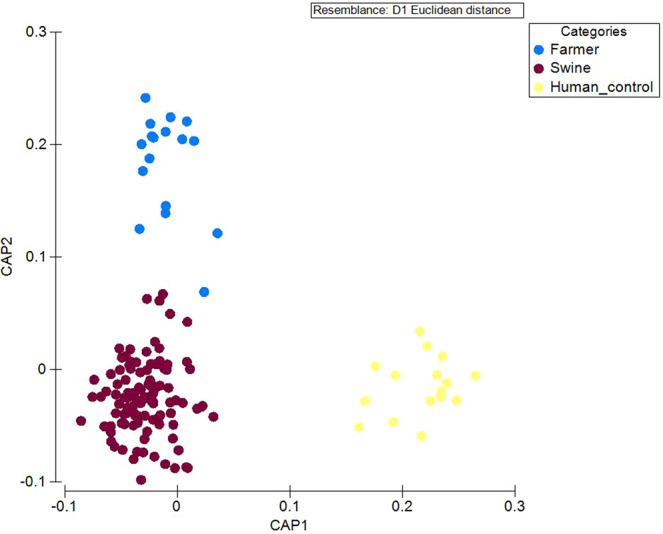
Faecal bacterial composition of swine, farmers and human control. The canonical analysis of principal coordinates (CAP) plot shows three distinct groups of bacterial composition were detected in different host (swine (n = 91), farmers (n = 17) and human control (n = 16)) based on T-RFLP.

A clear separation between the bacterial composition of swine and human control was observed in CAP1-axis of the CAP plot (Fig. 1). In comparison, swine and farmers were separated in CAP2-axis. The significance of the separation was statistically tested using PERMANOVA. Based on PERMANOVA, all three pairing including swine vs control, swine vs farmers and control vs farmers were statistically significant with P(MC) <0.05. While T-RFLP was useful in assessing the overall structure of the bacterial community, the method did not provide taxonomic information of the taxa present in the faecal samples collected. Thus, 40 samples (n_farmer_ = 16; n_swine_ = 16; n_human control_ = 8) were randomly selected for 16S pyrosequencing to elucidate the taxonomic composition of the three sampling groups (i.e. swine, human control and farmers).

### Metagenome analyses based on 16S pyrosequencing

A total of 304,658 raw reads were obtained from 16S pyrosequencing. The final dataset after trimming, quality filter and chimera removal consisted of 145,752 sequences. The coverage of the sequences ranged from 89–99% and the sequences were clustered into 3268 operational taxonomic units (OTUs). Venn diagram was constructed based on sequence abundance. Swine has the highest level of host specific taxa (n = 1555), followed by farmer (n = 771) and human control (n = 461) (Supplementary Fig. S1). A three times higher overlap in OTUs was observed between swine and farmer (n = 91) as compared to swine and human control (n = 30).

The faecal bacterial diversity, richness and evenness were determined by the Shannon-Weiner diversity index (*H*’), Simpson diversity index (1−λ) and Pielou’s evenness index (*J*’). Based on these alpha diversity indices, swine faecal sample had the highest richness and evenness (*H*’_swine_ = 3.52 ± 0.80; 1−λ*’*_swine_ = 0.90 ± 0.06; *J’*_swine_ = 0.67 ± 0.10), followed by farmers (*H’*_farmer_ = 3.20 ± 0.51; 1−λ*’*_farmer_ = 0.90 ± 0.06; *J’*_farmer_ = 0.63 ± 0.08) and human controls (*H’*_human control_ = 3.09 ± 0.44; 1−λ*’*_farmer_ = 0.90 ± 0.04; *J’*_human control_ = 0.58 ± 0.07) (Supplementary Fig. S2). A significant higher evenness was found in swine when compared to human control (F = 6.432, P = 0.019).

### Taxonomic composition of faecal samples obtained from swine, farmers and human controls

**Figure 2:**
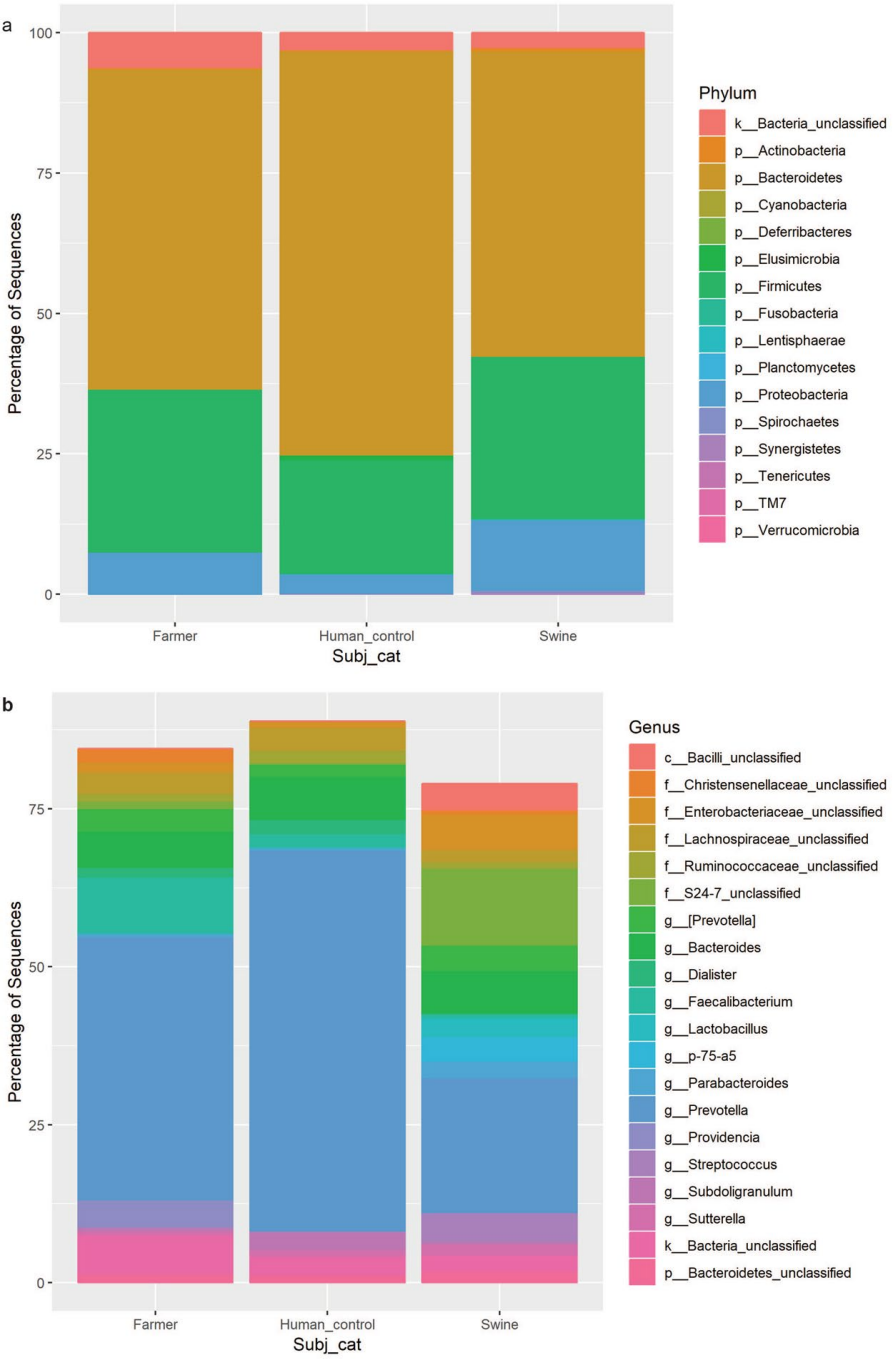
Relative abundance of faecal bacterial community. (**a**) Relative abundance of bacterial phyla in the faecal sample of swine, farmer and human control (**b**) Relative abundance of core bacterial genera in the faecal sample of swine, farmer and human control.

Bacteroidetes, Firmicutes and Proteobacteria made up >93% of the phyla detected in the faecal samples from all three groups. The relative abundance of Bacteroidetes was higher than Firmicutes in all three groups of samples (Figs. 2a,b), with the Firmicutes/Bacteroidetes ratios of 0.53, 0.46 and 0.19 for swine, farmer and human control, respectively.

At phylum level, farmer and swine harboured higher relative abundance of Firmicutes (29% in swine, 27% in farmers and 15% in human control) and Proteobacteria (12% in swine, 8% in farmers and 4% in human control) than human control (Fig. 2a). When the taxonomic composition was examined at genus level, *Prevotella* was the most dominant genus in all three groups (Fig. 2b). Both human samples (i.e. farmer and human control) showed higher level of *Lactobacillus* than swine. Overall, the top 20 bacterial genera were more dominant in both farmer and human control than in swine.

**Figure 3:**
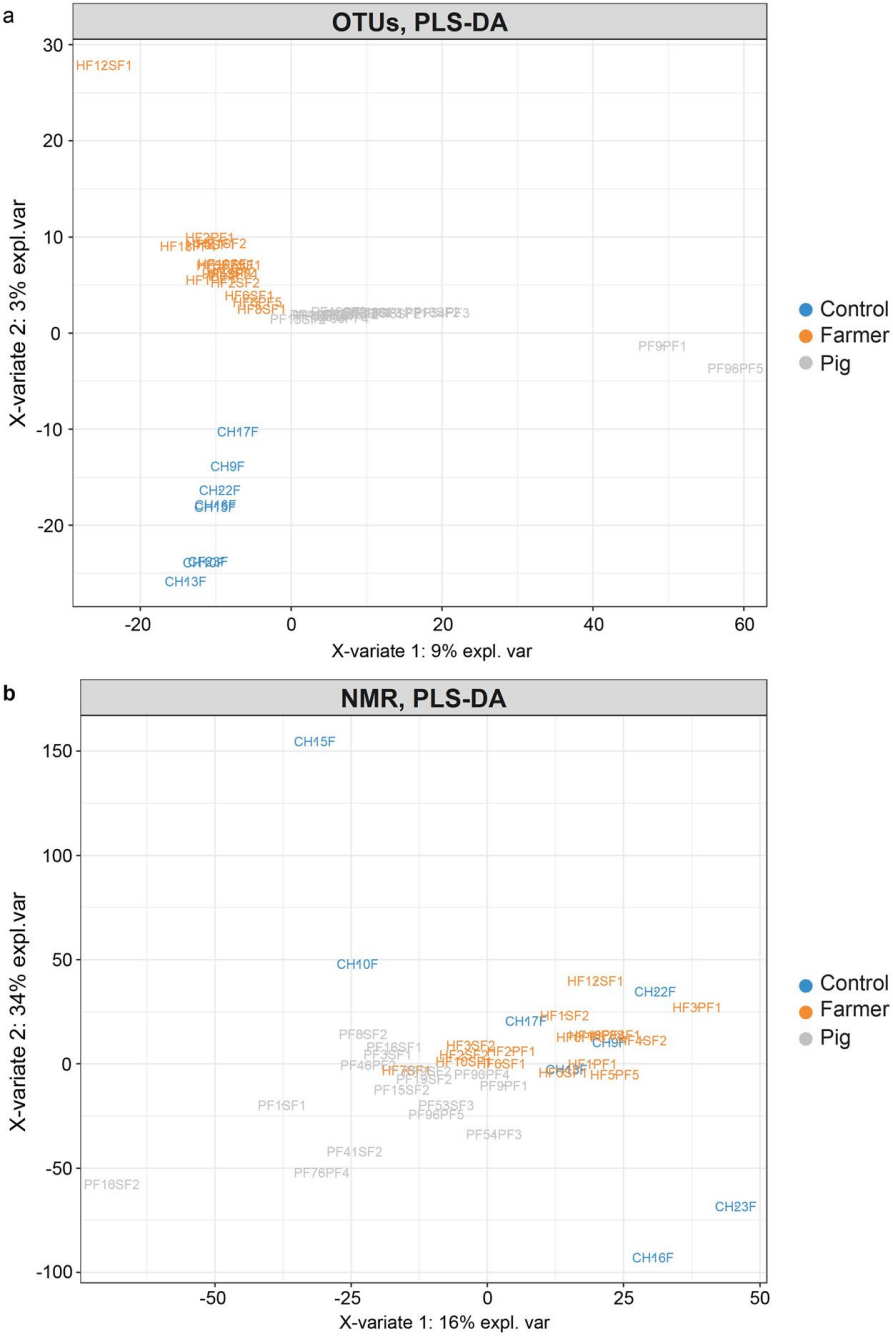
Faecal bacterial composition of swine, farmers and human control. (**a**) PLS-DA score plot showing the distribution of OTUs based on swine, farmer and human control. (**b**) PLS-DA score plot showing the metabonomes distribution of swine, farmer and human control.

Under PLS-DA, clear separation between humans (i.e. human control and farmer) and swine were observed in the axis X-variate 1 while human control clustered separately from swine in axis X-variate 2 (Fig. 3a). Permutation distance analysis of variance (PERMANOVA) with Euclidean distance showed that the metagenomics profiles of human control and swine were significantly different (Pseudo-T = 2.1386, P(MC) = 0.001). However, no significant difference was found between the faecal microbiota of farmers and swine (Pseudo T = 1.2942, P(MC) = 0.112) and between farmers and human controls (Pseudo-T = 1.2999, P(MC) = 0.110).

### Differentially expressed OTUs

Negative log binomial model was used to identify OTUs that differed significantly across host (Supplementary Fig. S3, Table 1). OTU0007 (*P. copri*), OTU0018 (*P. copri*), OTU0034 (*Dialister* spp.) and OTU0036 (*Faecalibacterium prausnitzii*) were highly expressed in farmers and human controls when compared to swine. OTU0002 (Enterobacteriaceae), OTU0011 (*Escherichia coli*), OTU0031 (unclassified bacteria under the class Bacilli) and OTU0044 (unclassified bacteria under Bacteroidales S47 family) were elevated in swine in comparison human control. Lastly, in comparison to farmers, OTU0031 and OTU0044, OTU0055 (*Streptococcus alactolyticus*) and OTU0062 (*Prevotella* spp.) were more prevalent in swine.

**Table 1:** Taxonomy of OTUs with significant expression in different groups of samples.

OTUs	Phylum	Class	Order	Family	Genus	Species
Otu0002^a^	Proteobacteria	Gammaproteobacteria	Enterobacteriales	Enterobacteriaceae	unclassified	unclassified
Otu0007^b,d^	Bacteroidetes	Bacteroidia	Bacteroidales	Prevotellaceae	Prevotella	copri
Otu0009^d^	Bacteroidetes	Bacteroidia	Bacteroidales	Prevotellaceae	Prevotella	copri
Otu0011^a^	Proteobacteria	Gammaproteobacteria	Enterobacteriales	Enterobacteriaceae	Escherichia	coli
Otu0014^d^	Firmicutes	Clostridia	Clostridiales	Lachnospiraceae	unclassified	unclassified
Otu0018^b,d^	Bacteroidetes	Bacteroidia	Bacteroidales	Prevotellaceae	Prevotella	copri
Otu0022^d^	Firmicutes	Clostridia	Clostridiales	Ruminococcaceae	Faecalibacterium	prausnitzii
Otu0031^a,c^	Firmicutes	Bacilli	Bacilli_unclassified	Bacilli_unclassified	unclassified	unclassified
Otu0034^b,d^	Firmicutes	Clostridia	Clostridiales	Veillonellaceae	Dialister	unclassified
Otu0036^b,d^	Firmicutes	Clostridia	Clostridiales	Ruminococcaceae	Faecalibacterium	prausnitzii
Otu0044^a,c^	Bacteroidetes	Bacteroidia	Bacteroidales	S24-7	unclassified	unclassified
Otu0055^c^	Firmicutes	Bacilli	Lactobacillales	Streptococcaceae	Streptococcus	alactolyticus
Otu0061^d^	Firmicutes	Erysipelotrichi	Erysipelotrichales	Erysipelotrichaceae	Catenibacterium	unclassified
Otu0062^c^	Bacteroidetes	Bacteroidia	Bacteroidales	Prevotellaceae	Prevotella	unclassified
Otu0068^d^	Firmicutes	Clostridia	Clostridiales	Ruminococcaceae	Faecalibacterium	prausnitzii
Otu0076^d^	Firmicutes	Clostridia	Clostridiales	Ruminococcaceae	Faecalibacterium	prausnitzii
Otu0113^d^	Firmicutes	Clostridia	Clostridiales	Ruminococcaceae	Faecalibacterium	prausnitzii
Otu0147^d^	Firmicutes	Clostridia	Clostridiales	Ruminococcaceae	Faecalibacterium	prausnitzii

^a^Upregulated in swine as compared to human control ^b^Upregulated in human control as compared to swine ^c^Upregulated in swine as compared to farmer ^d^Upregulated in farmer as compared to swine.

### Faecal metabolic profiles of humans and swine

**Figure 4:**
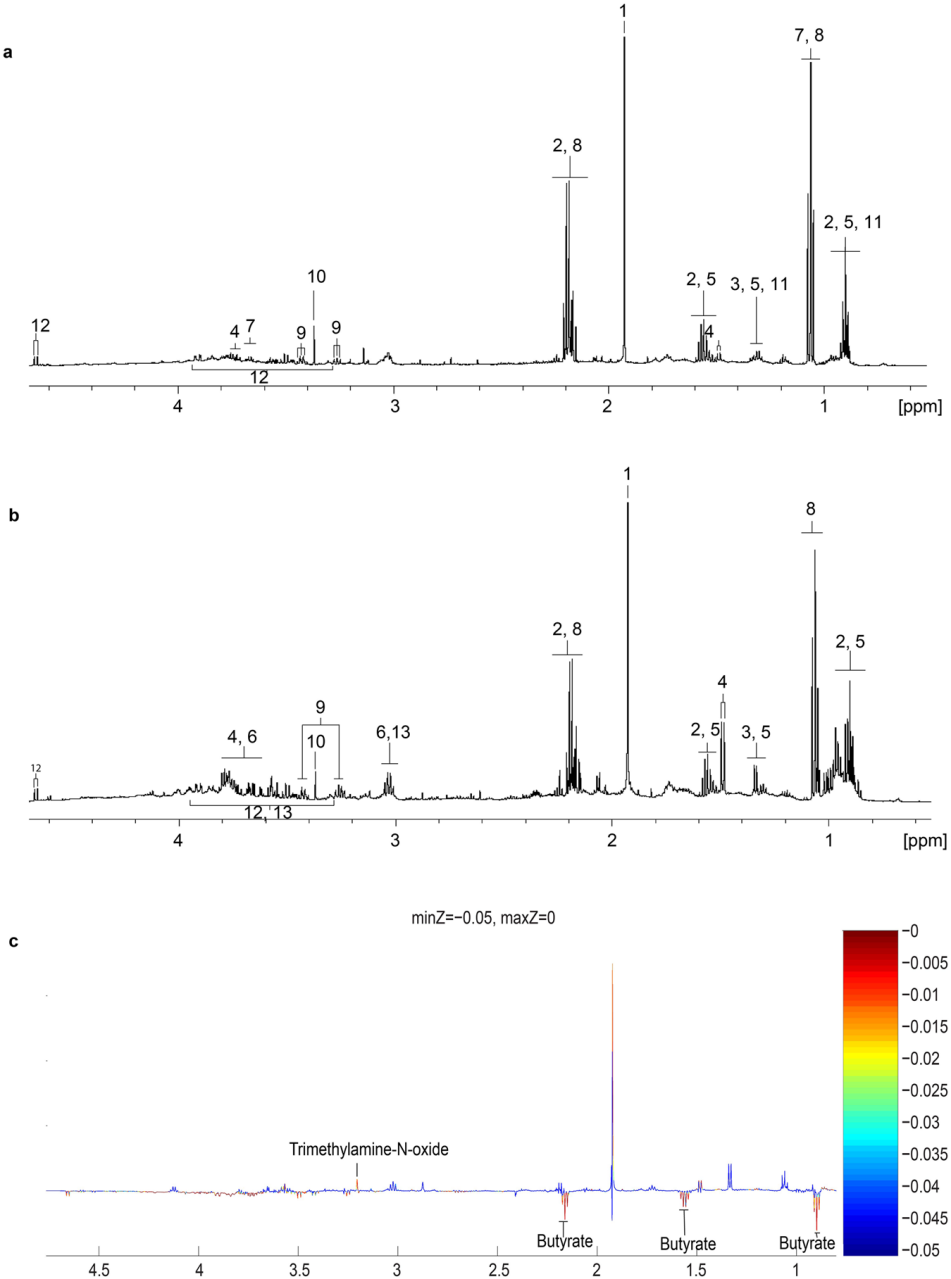
^1^H NMR spectroscopy showing the metabolites profiles of (**a**) human faecal sample, (**b**) swine faecal sample and the (**c**) covariance plot between the metabolites profiles of human control (upwards) and swine (downwards). Key as indicated in Table 2.

All metabolites reported in this study are listed in Table 2. Metabolites detected in the two groups of human samples (farmers and human controls) were identical, which included acetate, butyrate, lactate, alanine, lipids in VLDL, lipids in LDL, ornithine, ethanol, propionic acids, taurine, *Scyllo*-Inositol and β-glucose (Fig. 4a). The identity of the metabolites was validated by 2D-NMR spectroscopy. Except for ethanol, all other detected metabolites were also present in the swine faecal samples (Fig. 4b). PLS-DA and PERMANOVA were used to evaluate the differences in metabolic profiles between groups (Fig. 3b, Supplementary Table S1). Significant difference between metabonome was detected between human control and swine (Pseudo-T = 2.0793, P(MC) = 0.010), but not swine with farmers (Pseudo-T = 1.5397, P(MC) = 0.078), as well as farmers and human control (Pseudo-T = 1.2849, P(MC) = 0.175).

**Table 2:** ^1^H NMR peak assignments for identified metabolites^a^.

No.	Metabolites	Assignments	δ ^1^H, p.p.m (multiplicity)	Sample	References
1	Acetate	CH_3_	1.92 (s)	Human, Swine	HMDB, Merrifield *et al*. (2011)
2	Butyrate	CH_2_	2.14 (t)	Human, Swine	HMDB
		CH_2_	1.55 (tq)		
		CH_3_	0.88 (t)		
3	Lactate	CH_3_	1.32 (d)	Human, Swine	HMDB, Merrifield *et al*. (2011)
		CH	4.2 (q)		
4	Alanine	βCH_3_	1.49 (d)	Human, Swine	HMDB, Merrifield *et al*. (2011)
		αCH	3.79 (q)		
5	Lipids in VLDL	CH_3_CH_2_CH_2_C =	0.87 (t)	Human, Swine	HMDB, Merrifield *et al*. (2011)
		CH2CH2CH2CO	1.29 (m)		
		CH_2_CH_2_CO	1.57 (m)		
6	Ornithine	1/2 γCH_2_	1.72 (m)	Human, Swine	HMDB, Merrifield *et al*. (2011)
		1/2 γCH_2_	1.82 (m)		
		βCH_2_	1.93 (m)		
		δCH_2_	3.04 (t)		
		αCH	3.77 (t)		
7	Ethanol	CH_3_	1.19 (t)	Human	HMDB
		CH_2_	3.66 (q)		
8	Propionic acid	CH_3_	1.04 (t)	Human, Swine	HMDB, Merrifield *et al*. (2011)
		CH_2_COOH	2.17 (q)		
9	Taurine	N-CH_2_	3.26 (t)	Human, Swine	HMDB, Merrifield *et al*. (2011)
		S-CH_2_	3.43 (t)		
10	Scyllo-Inositol	CH	3.35 (s)	Human, Swine	HMDB, Merrifield *et al*. (2011)
11	Lipids in LDL	CH_3_(CH_2_)_n_	0.88 (t)	Human, Swine	HMDB, Merrifield *et al*. (2011)
		(CH_2_)_n_	1.28 (m)		
12	β-glucose	C2H	3.25 (m)	Human, Swine	HMDB, Merrifield *et al*. (2011)
		C4H	3.49 (m)		
		C5H	3.49 (m)		
		C3H	3.50 (m)		
		1/2 C6H_2_	3.88 (m)		
		1/2 C6H_2_	3.91 (d)		
		C1H	4.66 (d)		
13	Creatine	N-CH_3_	3.03 (s)	Swine	HMDB, Merrifield *et al*. (2011)
		N-CH_2_	3.94 (s)		

^a^Key: s = singlet, d = doublet, t = triplet, q = quartet, m = multiplet, tq = triplet of quartet, F = faecal, HMDB = Human Metabolome Database.

Significantly expressed metabolites were identified using permutation test and presented in the covariance plot (Fig. 4c). Among the detected metabolites, butyrate was found to be significantly elevated in swine as compared to human control (Fig. 4c). On the other hand, trimethylamine-N-oxide (TMAO) was over-expressed in human control in comparison to swine. No distinct metabolite was found to be differentially expressed between human control and farmers, as well as farmers and swine.

### Integration of gut microbial composition and metabolomics profiles

**Figure 5:**
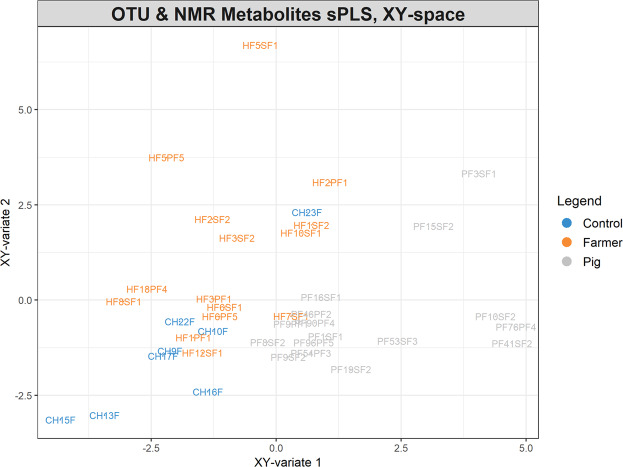
sPLS score plot showing the distribution of OTUs of all samples in relation to their metabolomics profiles.

**Figure 6:**
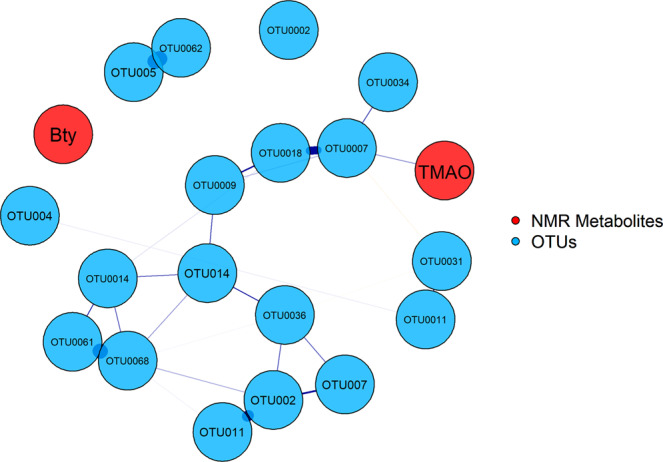
Correlation analysis of all significant OTUs with the two significant metabolites. The network analysis revealed the co-occurrence patterns of bacteria and the metabolites. The blue colour lines represent positive correlation and the thickness of the lines represents the distance.

The gut microbiota and metabolomics profiles were merged and projected using sPLs plot (Fig. 5). When both 16S gut microbial composition and faecal metabonome was considered together, a stronger clustering based on host species (human vs swine) was observed. A network analysis was further conducted to elucidate the association between the selected OTUs and metabolites (Fig. 6). Positive correlation was found between TMAO with OTU0007 (*P. copri*). However, butyrate which was significantly elevated in swine in comparison to human control was not correlated to any of the OTUs.

## Discussion

Direct contact is one of the major factors contributing to the transmission of pathogens between animals and humans. Close interaction between animals and humans can also increase the risk for horizontal transfer of antibiotic resistance genes in human microbiome^19^. Among the different types of “contact”, human-livestock contacts were the most common cause of zoonotic pathogens transmission^20^. Despite the importance, there is a lack of knowledge on the impact of close contact to the transfer of non-pathogenic commensals. Such notable lack of reports is striking, given the increasing recognition of the importance of both pathogenic and non-pathogenic members of microbiome in health^18^. In our previous study, we detected the presence of porcine-related *Enterococcus faecalis* (*E. faecalis*) in the gut of the humans and human-related strain in the gut of swine^21^. *E. faecalis* is a normal microbiota commonly found in the gut of humans and mammals. Consistent with this, porcine-related gentamicin-resistant *E. faecalis* were also reported in humans in Denmark in year 2010^22^. Such transmission not only present a health burden to the livestock and cause potential economic loss, but also poses a risk of subsequent reinfection in humans^23,24^.

In this study, we evaluated the impact of close human-swine interaction by integrating the results of 16S metagenomics and ^1^H-NMR-based metabolomics of faeces collected from swine, farmers and human control. Our result indicated the presence of host-specific gut microbiome between humans and swine (Fig. 1). The latter also showed higher alpha-diversity as compared to the former (Supplementary Fig. S2). In a parallel study by Sun *et al*.^25^, the faecal samples of swine farm workers were found to contain lower species diversity, while a clear division in faecal microbiota was observed between swine, farmers and the local villagers. Regardless, farmers harboured relatively more similar gut microbial community to swine in comparison to the human control, who has no direct contact with the livestock (Fig. 3a, Supplementary Fig. 1).

Overall, Firmicutes and Bacteroidetes were the predominant phyla found in all three groups of samples (swine, farmer and human control). The high prevalence of the two phyla (e.g. together attributed 85–90% of the total sequences) observed was consistent with previous reported microbiome assessments on humans and swine^2,15,26–28^. The Firmicutes/Bacteroidetes ratio is commonly related to the health status and diet of humans and swine^29–31^. Interestingly, we found a higher relative abundance of Bacteroidetes than Firmicutes in all three groups (Fig. 2a,b). Bacteroidetes involved in host’s metabolism possibly harvest energy from indigestible polysaccharides and produce short chain fatty acids (SCFAs). The microbiome in the guts of humans undergo a change in the relative abundance of the two major phyla at different stage of life^32^. An increase in Firmicutes/Bacteroidetes ratio was also reported in the gut microbiota of obese individuals^33–35^.

At genus level, a member of Bacteroidetes, *Prevotella* spp. dominated the faecal metagenome of all three groups of samples. One of the major species of *Prevotella* spp. is *Prevotella copri* (*P. copri*), which was previously reported to be positively associated with rheumatoid arthritis by favouring Th17 lymphocytes development and induced tissue damage in rheumatoid arthritis patients^36,37^. Apart from *P. copri*, a higher level of *Faecalibacterium prausnitzii* (*F. prausnitzii*) was detected in farmers. This bacterium was reported as one of the most abundant bacterial species in gut of healthy humans and animals, including swine^38^. *F. prausnitzii* is able to control gut epithelial cells metabolism, host immune response and produce important SCFA such as butyrate^39^. Butyrate is one of the major anti-inflammatory metabolites found in the gut. Previous studies had reported a decrease of *F. prausnitzi* in patient associated with psoriasis and inflammatory bowel disease such as Crohn’s disease, Coeliac disease and ulcerative colitis^40–42^. In the faeces of swine, we detected high prevalence of *Streptococcus alactolyticus*, which is a common commensal in animals such as dogs and swine but rarely detected in humans^43,44^. Nonetheless, zoonotic infection of *S. alactolyticus* infections in humans was previously reported^45,46^. Although the role of *S. alactolyticus* in gut was not clear, the bacterium is known to secrete functional metabolites such as amylase, galactosidase, β-glycoside hydrolase, acidic galactose, αgalactosidase, and urease^47^.

Gut microbiota plays an important role in maintaining the homeostasis of the host’s body and majority of the physiological contributions of gut bacteria are involved in fermentation and production of SCFAs such as acetate, propionate and butyrate. For instance, gut microbes can ferment complex carbohydrate in dietary fibre into SCFAs^48^. Although a shift in the faecal microbiota of farmers was observed, the overall faecal metabolites of the two groups of humans remained comparable. This shows that there is a high level of functional plasticity in the gut microbial community. In our study, two metabolites were found to be upregulated in specific sample group. A higher level of butyrate was found in the swine faeces. Although our integration study showed that the production of butyrate was not linked to any gut bacteria, an association of butyrate production with high abundance of Firmicutes was reported^49^. We speculate that the butyrate is produced collectively by a group of bacterial taxa and hence a linear relationship between the metabolite and bacterial OTUs is absent. Butyrate has been reported to associate with many health issues ranging from anti-inflammatory properties, host immunity and enhancement of intestinal barrier function^50^. On the other hand, *P. copri* was positively correlated with the level of TMAO found in the human control. TMAO is known to be a by-product of dietary choline digestion. Food rich in dietary choline include egg yolks and meats^51^. TMAO is vital for platelet responsiveness and thus plays a vital role in increasing the incidence of thrombotic events such as heart attack and stroke^52^. *P. copri* has been implicated in a number of autoimmune diseases such as colitis, inflammatory bowel disease and correlated with adverse cardiovascular effects due to the increase of TMAO as microbial by-product^53–55^. Our findings were concordant with previous studies by Scher *et al*.^56^ and Koeth *et al*.^51^ who reported that abundance of *P. copri* was correlated with the level of TMAO.

In summary, we showed that occupational contact between farmers and livestock may result to a bacterial community shift in human gut microbiome, as evident in the higher similarity in microbiome between farmers and swine than human control. Despite these changes, no substantial difference in the metabonome was detected between farmers and human control. The lack of effect may suggest that the changes are transient and can be compensated with the high functional plasticity of the gut bacteria. It is however possible that the health effect may only manifest under long-term exposure. As such, a long-term monitoring study of microbiome and health outcomes of farmers is warranted.

## Methods

### Samples collection

Seven swine farms (five farms located in the northern region coded as PF1, PF2, PF3, PF4 and PF5 and two farms located in central region coded as SF1 and SF2) located in the high-density swine farming areas in Peninsular Malaysia were sampled between August 2013 to December 2013. A total of 91 swine faecal samples were collected from the animals. All samples were collected under the supervision of a veterinarian from the Faculty of Veterinary Medicine, Universiti Putra Malaysia (UPM). Separately, 33 faecal samples were collected from swine farmers (n = 17) who worked in the seven participating farms and non-farmer human control group (n = 16). All swine farmers involved in this study have been working in the swine farms for at least two years. The human subjects were advised to defecate directly into the stool collection bottle or onto a clean surface and immediately transfer the faecal sample into the collection tube by using the scoop on the cap. Background information of the samples was inferred based on questionnaire as well as the observations and advices given by the attending veterinarian. The information included farm locations, farm hygiene practice, gender of the swine (male/female), body temperature and health condition of the swine (healthy/unhealthy). Physical examination (clinical signs, behavior and body temperature) of the swine was performed to determine their health status by the field veterinarian. Swine that presented with abnormal clinical signs, behavior and elevated body temperature were categorized as unhealthy. All the human subjects and swine were in healthy or asymptomatic condition during the sampling. All samples were transported on ice to Kuala Lumpur and stored at −80 °C at the earliest opportunity. This study was conducted following the guidelines as stated in the Code of Practice for Care and Use of Animals for Scientific Purposes as stipulated by UPM (UPM/IACUC/FYP- AUP-T006/), complied with the current guidelines for the care and use of animals, and was approved by the Animal Care and Use Committee (ACUC), Faculty of Veterinary Medicine, UPM. The human samples collection was approved by Medical Research Ethics Committee, University Malaya Medical Centre (UMMC-MREC) (Ethic committee/IRB reference number: 1010.41) and performed in accordance with the UMMC-MREC guidelines. Informed consent was obtained from all human subjects.

### Terminal-Restriction Fragment Length Polymorphism (T-RFLP)

DNA the faecal samples were extracted by using QIAamp DNA Stool Mini Kit (Qiagen, Hilden, Germany) according to manufacturers’ instruction. 16S rDNA amplifications were performed on the extracted DNA by using Universal primers 27F-FAM and 1492R-HEX as described in Chong *et al*.^57^. Briefly, both forward (27 F) and reverse primer (1492 R) were tagged with different fluorophores (i.e. FAM and HEX) via PCR. The PCR products were purified by using Wizard Genomic DNA purification kit (Promega, USA) and the purified DNA was digested with *Msp* I restriction enzyme (Promega, USA). The digested products were commissioned to a local commercial company for electrophoretic separation of restriction fragments. The resulting electropherograms were first processed with the Peak Scanner Software v1.0 (Life Technologies, USA). Subsequently, noise filtration, alignment and scoring were conducted using web-based T-REX program (http://trex.biohpc.org/). Peak alignment was carried out by binning the signals at a clustering threshold of 0.5 bp, starting from the smallest fragment length among all the T-RFLP profiles. The scoring of the peaks was recorded as peak area, and normalized by dividing the individual peak over the total peak area of each sample. The scoring datasheet was exported into PRIMER 7 & PERMANOVA (PRIMER-E Ltd, UK) for statistical analyses. Briefly, the beta diversity was assessed using Bray-Curtis Distance based canonical analysis of principal coordinates (CAP) and Permutational Multivariate Analysis of Variance (PERMANOVA).

### Amplicons -Next-Generation Sequencing (16S-NGS)

Forty samples including 16 swine, 16 farmers and 8 human control were selected for 16S-NGS. The 16S rRNA genes fragments from variable V3 regions were amplified using primer set 27 F (GAGTTTGATCMTGGCTCAG) and 518 R (WTTACCGCGGCTGCTGG) containing sample specific barcodes. Amplicon pyrosequencing was performed by Macrogen Inc. (Seoul, South Korea) using Roche 454 GS-FLX system (Roche, NJ, USA). The pyrosequencing produced a total of 808,275 sequence reads with an average read length of 374 bp. The sequences obtained were processed using Mothur software (v.1.34.3)^58^ according to the 454 SOP (http://www.mothur.org/wiki/454_SOP). In brief, the raw sequences were first processed by “sff.multiple” command. The sequences were denoised and filtered by removing sequence shorter than 250 bp and longer than 550 bp. In addition, maximum homopolymer count was set at 6 bp while the maximum allowable differences in primer and barcode sequences were set at 2 bp. The sequences were aligned to SILVA-compatible alignment reference database (Version 132). Sequences which were poorly aligned and overhangs at the both ends were removed so that the sequences overlapped at the same region. Unique sequences were screened and chimeric and ambiguous sequences classified to unrelated taxon were removed by using “chimera.uchime” and “remove.lineage” commands. The dataset was clustered into OTU by using 97% cut-off. The final aligned dataset contained 17,660 unique sequences. Alpha diversity was assessed with Shannon diversity index, Simpson index, Pielou’s evenness. The “DIVERSE” option in the PRIMER 7 data analyses packages (PRIMER-E Ltd, UK) was used to obtain the alpha diversity index. The beta diversity among the samples were elucidated using Partial Least Square - Discriminant Analysis (PLS-DA) and PERMANOVA. Prior to the analysis, the data was ‘regularised log’ transformed. PLS-DA implemented in the mixOmics R package^59^ was used to visualise the separation between different groups of samples while the compositional differences was compared using PERMANOVA. Separately, differentially expressed OTUs were identified based on negative binomial distribution using DESeq. 2 R package^60^.

### Sample preparation and ^1^H NMR spectroscopic analysis

Faecal samples were processed by using the NMR buffer [1 mM of 3-(trimethylsilyl) propionate (TSP) and 3 mM sodium azide (D_2_O: H_2_O, v/v, 8:2; pH 7.4)]. TSP was used as a reference for chemical shift. For each sample, 0.05 g of faecal matter was homogenized and vortexed in one ml of NMR buffer. The mixture was sonicated for 30 min and centrifuged at 14,000 rpm for 10 min. Six hundred µl of supernatant were transferred to 5 mm-diameter NMR tubes (Norell, USA). The processed samples were stored at −80 °C until analysis.

A standard 1-dimensional (1-D) ^1^H NMR spectrum was acquired by using Bruker AVIII 600 MHz spectrometer (Bruker Biospin, Fallenden, Switzerland) with a 5 mm PABBO BB probe operating at 600.17 MHz. The field frequency was locked on the D_2_O solvent and water peak suppression was performed during RD of 2 s and mixing time (t_m_) of 10 s. In addition, 2-D NMR using ^1^H-^1^H correlation spectroscopy (COSY) and ^1^H-^1^H J-resolved (JRES) were performed on selected representative samples to assist metabolite identification.

The NMR spectra were manually phase- and baseline-corrected using Bruker TopSpin 4.0.6 and imported into MATLAB (version 2014b). All the spectra were referenced to the TSP resonance at δ 0.00. The spectra were digitized into data point using in-house developed MATLAB script (O. Cloarec, Imperial College London). The region containing noise (δ 0.0–0.5 and δ 9.2–10.0) and water resonance (δ 4.5–6.5) were removed. Spectra normalization was performed and the regions with TSP peaks, water presaturation imperfection and the end regions containing only noise were removed. PLS-DA was used to illustrate the relationship between groups. The significance and validity of statistical differences were calculated using permutation test (number of permutations = 1000). Covariance plots were generated to visualize the significance of each metabolite from the permutation test. The colour scheme projected onto the spectrum indicate the significance of the metabolites. Blue indicating to no significant difference (P > 0.05 confidence level) and red indicating significant difference (P < 0.01 confidence level). The relative concentrations of the significant metabolites were further calculated by using in-house developed MATLAB script (O. Cloarec, Imperial College London).

### Linking faecal metabolites with gut microbiota composition

The integration and visualization of OTUs and metabonomes was performed using sparse partial least squares (sPLS) regression method implemented in R mixOmics package. sPLS allows the integration of heterogeneous omics data from the same set of samples, OTUs (matrix X) and metabonomes (matrix Y). The relationship was projected using sPLS plot and network diagram.

### Declarations of ethical approval and consent to participate

This study was approved by the Animal Care and Use Committee (ACUC), Faculty of Veterinary Medicine, UPM and conducted according to the guidelines as stated in the Code of Practice for Care and Use of Animals for Scientific Purposes as stipulated by UPM (UPM/IACUC/FYP- AUP-T006/). The human samples collection was approved by Medical Ethics Committee, University Malaya Medical Centre (Ethic committee/IRB reference number: 1010.41) and performed with the informed consent of human subjects.

## Supplementary information

Supplementary information.

Supplementary information 2.

## Data Availability

The datasets generated during and/or analysed during the current study are available from the corresponding author on reasonable request.
