# Supplementary material for: Comparative assessment of faecal microbial composition and metabonome of swine, farmers and human control
Source: Sci Rep. 2020 Jun 2;10:8997. doi: 10.1038/s41598-020-65891-4 (PMC7265441; doi:10.1038/s41598-020-65891-4)
Supplement: Supplementary file 1 — Supplementary information. [file 41598_2020_65891_MOESM1_ESM.pdf]

Comparative assessment of faecal microbial composition and metabonome of swine, farmers and human control

Shiang Chiet Tan[1], Chun Wie Chong[2,3], Ivan Kok Seng Yap[4], Kwai Lin Thong[5], Cindy Shuan Ju Teh[6*]

1 Institute of Biological Science, Faculty of Science, University of Malaya, 50603 Kuala Lumpur, Malaysia

2 School of Pharmacy, Monash University Malaysia, Jalan Lagoon Selatan, 47500 Bandar Sunway, Malaysia

3 Centre for Translational Research, Institute for Research, Development and Innovation (IRDI), International Medical University, 57000 Kuala Lumpur, Malaysia

4 Sarawak Research and Development Council, 11th Floor LCDA Tower, The Isthmus, Off Jalan Bako, 93050 Kuching, Sarawak, Malaysia

5 NANOCAT Research Centre, University of Malaya, 50603 Kuala Lumpur, Malaysia.

6 Department of Medical Microbiology, Faculty of Medicine, University of Malaya, 50603 Kuala Lumpur, Malaysia

* Corresponding author:

Cindy Shuan Ju Teh

Email: cindysjteh@um.edu.my

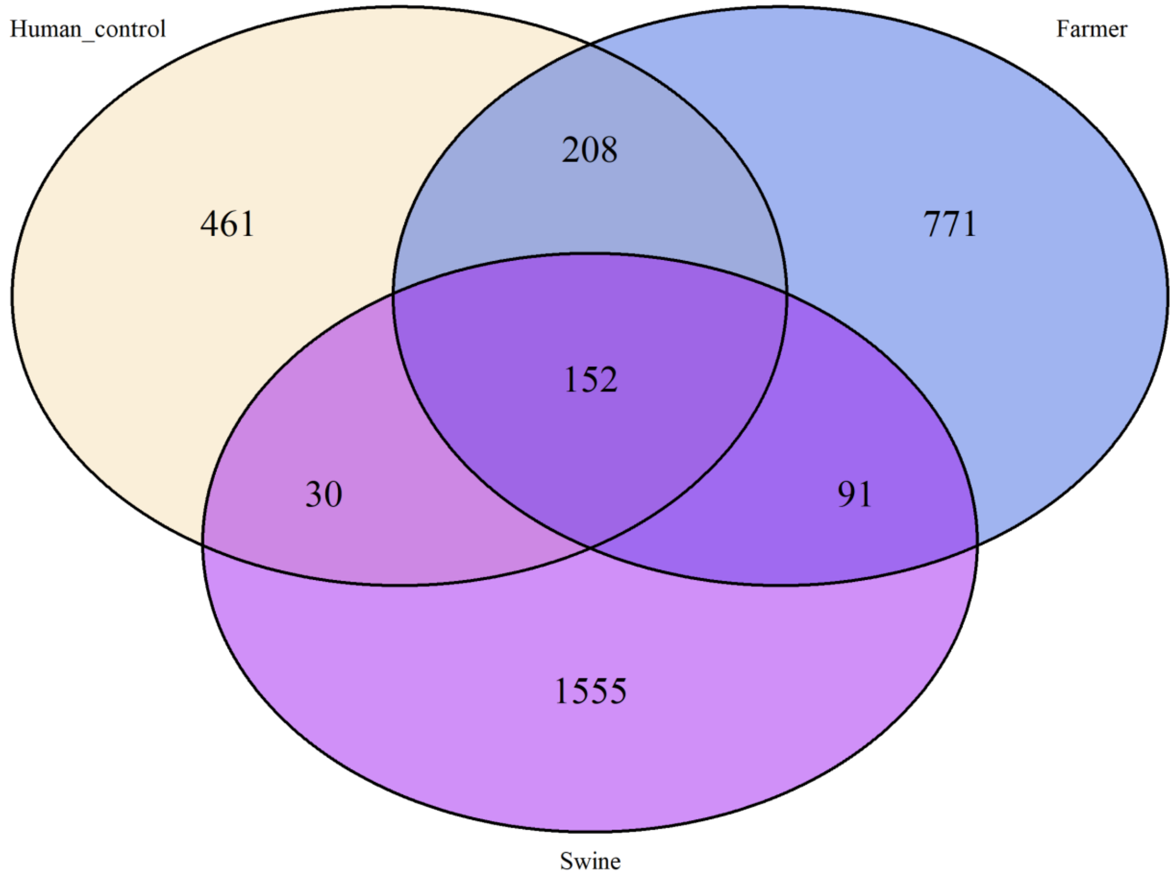

Supplementary Fig. S1 Venn diagram showing the number of unique and shared OTUs among the human controls, farmers and swine.

A

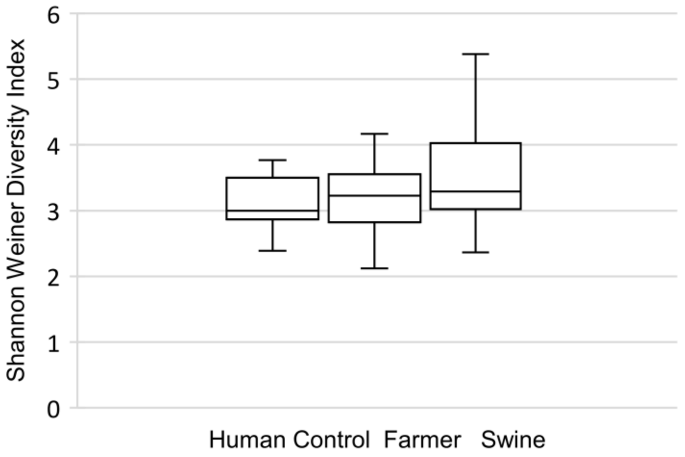

B

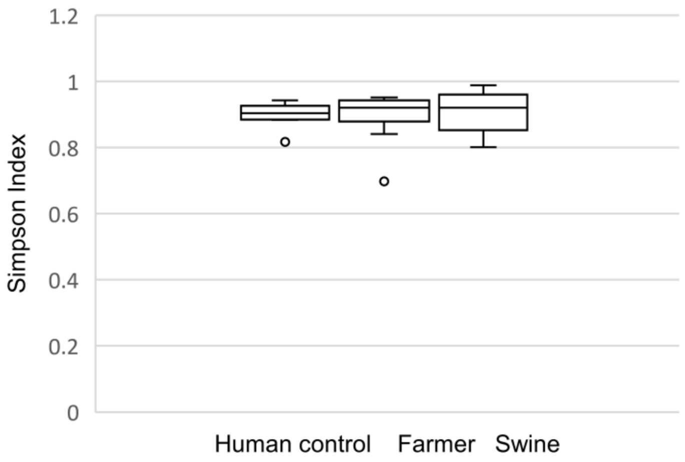

C

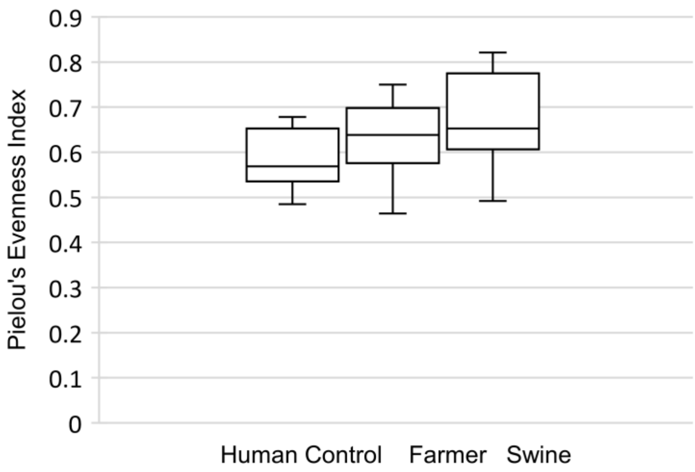

Supplementary Fig. S2. Boxplot of richness and evenness estimators.
(A) Shannon-Weiner diversity index; (B) Simpson diversity index; (C) Pielou’s evenness index.

A

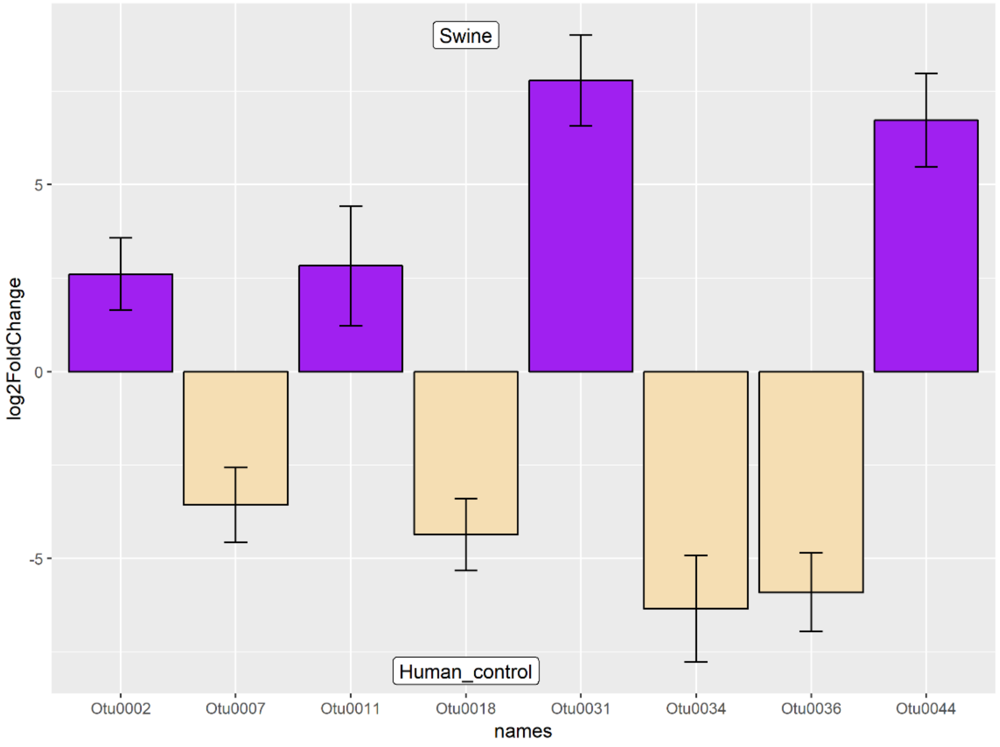

B

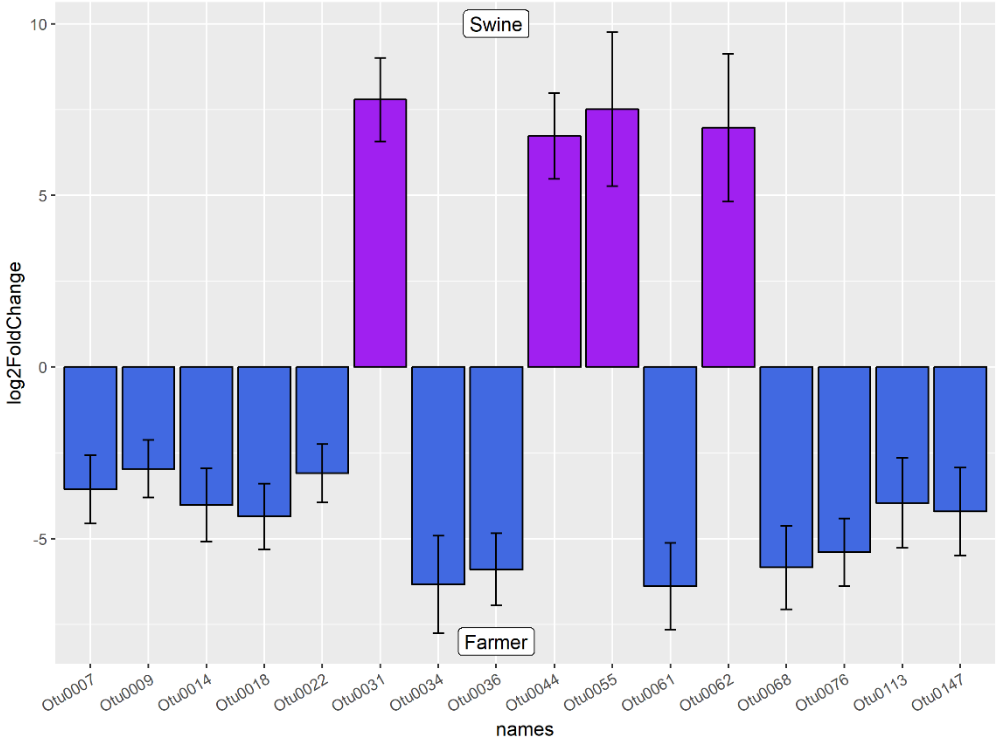

Supplementary Fig. S3. Log2 fold changes of respective OTUs in different group of samples, (A) between human control and swine and (B) between farmers and swine

Supplementary Table S1. Statistical significant difference of metabolites profiles of different groups of samples

	SIMCA			PRIMER6		
	PLS-DA			PERMANOVA		
Groups	R2X	R2Y	Q2	t	p(perm)	p(MC)
Farmer vs control	-	-	-	1.2849	0.146	0.175
Farmer vs Swine	-	-	-	1.5397	0.072	0.078
Control vs Swine	0.551	0.994	0.771	2.0793	0.007	0.010
