# Supplementary material for: Comparative assessment of faecal microbial composition and metabonome of swine, farmers and human control
Source: Sci Rep. 2020 Jun 2;10:8997. doi: 10.1038/s41598-020-65891-4 (PMC7265441; doi:10.1038/s41598-020-65891-4)
Supplement: Supplementary file 2 — Supplementary information 2. [file 41598_2020_65891_MOESM2_ESM.docx]

**Supplementary Information**

**Supplementary Fig. S1** Venn diagram showing the number of unique and shared OTUs among the human control, farmer and swine.

**Supplementary Fig. S2.** Boxplot of richness and evenness estimators.
(A) Shannon-Weiner diversity index; (B) Simpson diversity index; (C) Pielou’s evenness index

**Supplementary Fig. S3.** Log2 fold changes of respective OTUs in different group of samples, (A) between human control and swine and (B) between farmers and swine

**Supplementary Table S1.** Statistical significant difference of metabolites profiles of different groups of samples
